# Long Term Mortality And Risk of End-Stage Renal Disease Following Acute Rrt in Icu Patients

**DOI:** 10.1186/2197-425X-3-S1-A11

**Published:** 2015-10-01

**Authors:** R Lohse, MB Damholt, J Wiis, A Perner, T Lange, M Ibsen

**Affiliations:** Copenhagen University Hospital - Rigshospitalet, Department of Intensive Care 4131, Copenhagen, Denmark; Copenhagen University Hospital, Rigshospitalet, Department of Nephrology 2132, Copenhagen, Denmark; Copenhagen University Hospital, Rigshospitalet, Department of Intensive Care 4131, Copenhagen, Denmark; University of Copenhagen, Section of Biostatistics, Copenhagen, Denmark

## Introduction

In the intensive care unit (ICU) the need for acute renal replacement therapy (aRRT) is associated with high mortality and risk of end-stage renal disease (ESRD). We investigated long term mortality and progression to ESRD in ICU patients requiring aRRT and factors associated to these.

## Methods

Retrospective analysis of all adult patients admitted to the general ICU, Rigshospitalet, from 1/1-2005 to 31/12-2012, identified through the ICU database, excluding chronic dialysis patients. ESRD was defined as the need of dialysis >90 days or kidney transplant.

## Results

Of 5766 patients included, 931 (16%) received aRRT, 4762 (83%) did not receive any RRT and 73 (1%) only received aRRT during a later ICU admission. The 90-day mortality was 55% for aRRT patients and 22% for those not requiring aRRT (p < 0.001). The median (IQR) age was 61 (49 - 68) for aRRT 90-day survivors and 65 (55 - 73) for aRRT non-survivors (p < 0.001). The median SAPS II score was 53 (43 - 64) for aRRT 90-day survivors and 61 (49 - 74) for aRRT non-survivors (p < 0.001). Characteristics of aRRT patients developing ESRD and those that did not, are displayed in tables 1-3. The 7-year risk of ESRD for patients surviving 90 days after admission was 10% (7 - 14) for aRRT patients as compared to 0.5% (95% CI 0.3-0.9) for those not receiving aRRT (p < 0.001).

**Table 1:** Baseline characteristics.

	**Data available**	**90 day survivors with ESRD (N = 38)**	**90 day survivors without ESRD (N = 404)**	***P***		
Age (years), median (IQR)	442	61	(52.0 - 70.0)	61	(49 - 68)	0.400
Gender (male)	442	25	(65.8%)	271	(67.1%)	0.999
Days in the ICU, median (IQR)	442	7.2	(3.6 - 15.6)	10.9	(5.7 - 20.9)	0.087
SAPS II score (points), median (IQR)	427	56	(41 - 68)	53	(43 - 64)	0.39
APACHE II score (points), median (IQR)	427	30	(25 - 35)	26	(21 - 31)	0.009
First SOFA (points), median (IQR)	417	11	(8 - 15)	12	(9 - 14)	0.861
MAX SOFA (points), median (IQR)	418	12	(9 - 15)	13	(11 - 16)	0.377
Vasopressor treatment	442	24	(63.2%)	297	(73.5%)	0.279
Mechanical ventilation	442	30	(78.9%)	371	(91.8%)	0.040

**Table 2:** Preexisting Comorbidity.

	**Data available**	**90 day survivors with ESRD (N = 38)**	**90 day survivors without ESRD (N = 404)**	***P***		
Chronic kidney disease (non-dialysis)	442	23	(60.5%)	90	(22.3%)	< 0.001
Hypertension	442	14	(36.8%)	119	(29.5%)	0.505
Diabetes	442	6	(15.8%)	56	(13.9%)	0.784
Congestive heart failure	442	4	(10.5%)	39	(9.7%)	0.993
Peripheral vascular disease	442	9	(23.7%)	82	(20.3%)	0.734
Cerebrovascular disease	442	3	(7.9%)	36	(8.9%)	0.968
Malignant neoplasm	442	12	(31.6%)	83	(20.5%)	0.180
Ischemic heart disease	442	4	(10.5%)	29	(7.2%)	0.783
Chronic obstructive pulmonary disease	442	4	(10.5%)	50	(12.4%)	0.928

**Table 3:** Primary diagnosis during ICU stay.

	**Data available**	**90 day survivors with ESRD (N = 38)**	**90 day survivors without ESRD (N = 404)**	***P***		
Sepsis	442	7	(18.4%)	112	(27.7%)	0.466
Other infectious diseases	442	6	(15.8%)	20	(5.0%)	0.025
Endocrinological diseases	442		(0.0%)	3	(0.7%)	0.868
Cardiovascular diseases	442	3	(7.9%)	43	(10.6%)	0.869
Respiratory diseases	442	9	(23.7%)	66	(16.3%)	0.514
Gastrointestinal or liver diseases	442	2	(5.3%)	11	(2.7%)	0.675
Trauma or poisoning	442	4	(10.5%)	38	(9.4%)	0.975
Other	442	7	(18.4%)	111	(27.4%)	0.483

## Conclusions

The aRRT patients surviving 90 days were younger and less severely ill at ICU admission as compared to the non-survivors. The survivors developing ESRD had more frequently preexisting chronic kidney disease and higher APACHE II scores as compared to those who did not develop ESRD. aRRT patients had increased risk of ESRD up to 7 years after ICU admission, emphasizing the KDIGO recommendations to follow up AKI patients due to their increased risk for CKD.

